# Allelic and genotype frequencies of major CYP2B6 polymorphisms in the Pakistani population

**DOI:** 10.1002/mgg3.1527

**Published:** 2021-02-18

**Authors:** Sagheer Ahmed, Saeed Khan, Kholood Janjua, Imran Imran, Arif Ullah Khan

**Affiliations:** ^1^ Department of Basic Medical Sciences Shifa College of Pharmaceutical Sciences Shifa Tameer‐e‐Millat University Islamabad Pakistan; ^2^ Dow International Medical College Dow University of Health Sciences Karachi Pakistan; ^3^ Shifa Clinical Research Center Shifa International Hospital Islamabad Pakistan; ^4^ Department of Pharmacology Faculty of Pharmacy Bahauddin Zakariya University Multan Pakistan; ^5^ Riphah Institute of Pharmaceutical Sciences Riphah International University Islamabad Pakistan

**Keywords:** CYP2B6, Pakistani population, pharmacogenetics, polymorphisms

## Abstract

**Background:**

Cytochrome P450 (CYP2B6) is an important enzyme that metabolizes about 3.0% of therapeutic drugs. Drugs metabolized mainly by CYP2B6 include artemisinin, bupropion, cyclophosphamide, efavirenz, ketamine, and methadone. The genetic polymorphisms in the *CYP2B6* gene have earlier been studied in many populations, but the data are lacking for the Pakistani population. This research study aimed to determine the frequencies of the three of the most important variant alleles and genotypes of the *CYP2B6* gene in the Pakistani population.

**Methods:**

Blood was withdrawn from healthy volunteers after taking informed consent. DNA was extracted using commercial kits, and allelic and genotype frequencies were determined after PCR amplification followed by restriction fragment length polymorphism (RFLP) and gel electrophoresis.

**Results:**

Our results show a minor allele frequency of 33.8% for CYP2B6*6, 25.8% for CYP2B6*4, 6.5% for CYP2B6*3, whereas wild‐type genotype frequency was 48.57% for CYP2B6*6, 59.79% for CYP2B6*4, and 90.20% for CYP2B6*3. A significant interethnic variation was also observed.

**Conclusions:**

Our results suggest that the frequency of poor metabolizers of CYP2B6, especially *6 variant, is significant enough in the Pakistani population to be given an important consideration when drugs metabolized by this enzyme are prescribed.

## Graphical abstract

This study shows that a significant portion of the Pakistani population possesses at least one abnormal activity allele of the CYP2B6 gene, especially the frequency of the CYP2B6*6 variant is significant enough in the Pakistani population to be given an important consideration when drugs metabolized by this enzyme are prescribed.

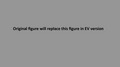

## 1 INTRODUCTION

The cytochrome P450 2 (CYP2) family, one of the major families of the cytochrome enzymes, has a large number of subfamilies that are aggregated together in the form of clusters in the genome. On chromosome 19 of the human cell, the *CYP2B* gene resides in one such cluster of six subfamilies, including the *CYP2A*, *CYP2B*, *CYP2F*, *CYP2G*, *CYP2S*, and *CYP2 T* genes (Simonsson et al., 2003). The *CYP2B6* gene (OMIM accession ≠ * 123930) possesses nine exons that encode for 491 amino acids containing protein (Yamano et al., 1989). The human *CYP2B6* gene has two known loci: the functional *CYP2B6* and its non‐functional pseudogene *CYP2B7*, located in the center of the *CYP2A18P* locus inside a 112 kb block (Hoffman et al., 2001; Miles et al., 1990; Yamano et al., 1989). CYP2B6 enzyme is involved in the metabolic activation and inactivation of several small molecule inhibitors, including anticancer (Chang et al., 1993; Granvil et al., 1999; Roy et al., 1999), antimalarial (Simonsson et al., 2003), and antidepressant drugs (Faucette et al., 2000; Hesse et al., 2000). Although the total fraction of the CYP2B6 enzyme is small as compared to the total hepatic P450 family, it still metabolizes a vast majority of pharmaceutical drugs (Mo et al., 2009; Wang & Tompkins, 2008). In addition to 7‐8% of the marketed pharmaceutical drugs, it also metabolizes certain exogenous and endogenous substances such as nicotine (Schoedel et al., 2003) and testosterone (Rosenbrock et al., 1999), in conjunction with other cytochrome enzymes.

Single‐nucleotide polymorphisms (SNPs) in the *CYP2B6* gene affect the expression and enzyme activity of the translated protein, resulting in significant differences in the pharmacokinetics of CYP2B6‐metabolized drugs among individuals and races, in turn, leading to variations in efficacy and toxicity (Aurpibul et al., 2012; Desta et al., 2007; Nyakutira et al., 2008). Several important variants of *CYP2B6* alleles: CYP2B6*2 (C64T), *3 (C777), *4 (A785G), *5 (C1459T), *6 (G516T and A785G), *7 (G516T, A785G, and C1459T) (Lang et al., 2001), *8 (A415G), and *9 (G516T) (Lamba et al., 2003) have been discovered, on top of the wild‐type CYP2B6*1 allele. About 38 protein variants of the highly polymorphic *CYP2B6* gene have been identified until now (http://www.cypalleles.ki.se/cyp2b6.htm), (Zanger & Klein, 2013). Gene variants of the enzyme result in allele, substrate, and expression dependent functional changes (Turpeinen & Zanger, 2012). Expression of the CYP2B6 enzyme is also affected by certain drugs like phenobarbital, which, by interacting with the phenobarbital response element in the *CYP2B6* gene, increases its expression (Pascussi et al., 2003; Sueyoshi et al., 1999).

Numerous other studies have reported that gene variations that result in changes in the expression of the CYP2B6 enzyme also result in altered drug responses (Coller et al., 2002; Hesse et al., 2000; Lerman et al., 2002). Studies have also shown that there are significant differences in the amount of enzyme and its activity among different individuals (Code et al., 1997; Coller et al., 2002; Ekins et al., 1998; Stresser & Kupfer, 1999). Differences in gene expression levels and splice variants have been found among several ethnic groups and have also been considered to be gender‐based (Lamba et al., 2003). For example, CYP2B6*4 variant (rs2279343, NC_000019.9:g.41515263A>G) but not CYP2B6*3 (rs45482602, NG_007929.1:g.23052C>A) has been shown to result in enhanced expression and variably increased/decreased activity of the enzymes (Gadel et al., 2015). Another SNP, CYP2B6*6 (rs3745274, NC_000019.9:g.41512841G>T) was alone responsible for aberrant splicing, resulting in high‐splice variant 1 and low‐CYP2B6 expression phenotype (Hofmann et al., 2008). In recent years, researchers have conducted a lot of studies investigating CYP2B6*6, and have found it to be associated with enhanced plasma concentrations of certain drugs (Aurpibul et al., 2012).

Pakistan is a culturally diverse country, but little is known about the distribution of *CYP2B6* genetic polymorphism in this country of over 200 million people. Various parts of the country possess a unique lifestyle, diverse genetic background, dietary habits, culture, and geographical environment. Several SNPs are found in the *CYP2B6* gene in addition to some copy number variable. However, only a few might alter the enzyme activity or associated with certain diseases. Therefore, we specifically investigated samples drawn from six of Pakistan's most populous ethnic groups located in distinct geographical locations and found out frequencies of three relevant polymorphisms (CYP2B6*6, *4, and *3) and then compared them with previous findings in other populations.

## 2 MATERIALS AND METHODS

### 2.1 Ethical compliance

This study was approved by the Institutional Review Board and Ethics Committee of Shifa Tameer‐e‐Millat University, Islamabad, Pakistan. Written Informed consent was obtained from all participating individuals.

### 2.2 Sample collection and DNA extraction

Study cohort of 490 healthy human volunteers comprised of six major ethnicities of Pakistan, including Punjabis, Pathan, Sindhi, Balochi, Seraiki, and Urdu Speaking. Ethnicity was self‐reported. Five milliliters of venous blood drawn into sterile tubes containing EDTA as an anti‐coagulant were stored at 4°C. Genomic DNA was isolated using Gene Jet Genomic DNA extraction Kit (ThermoScientific) and was quantified using 1% agarose gel electrophoresis. Isolated genomic DNA was stored at −20°C until further processing.

### 2.3 Genotyping

CYP2B6*6, CYP2B6*4, and CYP2B6*3 were genotyped using polymerase chain reaction (PCR) followed by restriction fragment length polymorphism (RFLP) as described previously (Zakeri et al., 2014). All amplifications were carried out in 25 μl reactions including 1 μl of the genomic DNA template. The primers were contained 10 mM Tris‐HCl (pH 8.3), 50 mM KCl, 2 mM MgCl2, each of the four deoxynucleotide triphosphates at a concentration of 125 μM, and 0.2 U of Taq polymerase (Invitrogen, Carlsbad, CA). The PCR program was 94°C for 5 min, followed by 30 cycles of 94°C for 1 min, 60°C for 1 min and 72°C for 1 min, with a final extension step of 72°C for 5 minutes. Digestions were carried out in 20 μl reactions containing 10 μl of PCR fragments according to the manufacturer's instructions. The DNA fragments were then electrophoresed on agarose gels. The primers and restriction enzymes used for each SNP are given in Table 1.

**Table 1:** Primer sequences and restriction enzymes used in the study

Allele	Primer Sequence	Restriction Enzyme	Product Size (bp)
CYP2B6*3	F: TCACCACCCCTTCTTTCTTG	HaeII	C:329 + 157 = 486
	R: AATTCCTTCCTCAGCCAGTC		
CYP2B6*4	F: GACAGAAGGATGAGGGGAGGAA	StyI	A:116 + 56 + 171 + 297 = 640
	R:CTCCCTCTGTCTTTCATTCTGT		
CYP2B6*6	F: ATAGCTGTGTTGCCTGGG	BseNI	T: 431 + 102: 533
	R: TTCTCGTGTGTTCTGGGTG		

### 2.4 Statistical analysis

Data were compiled according to the genotype and allele frequencies estimated from the observed numbers of each specific allele. The frequency of each allele and genotype in our samples is given together with the 95% confidence interval. The confidence interval for proportions was calculated using the formula (CI = *p* ± (1.96 × SE), SE = qrt [*p*(1 − *p*)/*n*], *p* = proportion, *n* = sample size). Chi‐squared test and *p* values were calculated using observed and expected frequencies as per the Hardy–Weinberg equation.

## 3 RESULTS

### 3.1 Alleleic and genotype frequency of CYP2B6*6

Frequencies of CYP2B6*6 alleles in the Pakistani population are shown in Table 2. The frequency of the major allele was 66.19% and of minor allele was 33.80%. The major allele was found slightly less prevalent in Punjabi and Baloch ethnic groups at 60.41% and 61.11%, respectively, while Seraiki samples displayed the lowest major allele frequency at 52.77%. Pathan and Urdu populations showed higher major allele frequencies, while the Sindhi Population displayed the highest major allele frequency among Pakistani ethnic groups. The frequency of the GG genotype was 48.57%, GT was 35.23%, and TT was 16.19% in the Pakistan population. Ethnic Punjabi Population showed a lower frequency of wild‐type genotype at 45.83%, while Pathan, Sindhi, Balochi, and Urdu ethnicities had a comparatively higher prevalence of wild‐type genotype. Sindhi Population showed the highest frequency of wild‐type genotype (GG) at 69.74%. Seraiki Population displayed the lowest prevalence of wild‐type genotype at only 22.22% (Table 3).

**Table 2:** CYP2B6*6 (rs3745274) allele frequencies in the Pakistani population

Ethnicity	No	Allele G % (CI)	Allele T % (CI)	Chi‐Square Statistic	*p*‐value
Pakistan	490	66.19 (62.0‐70.38)	33.80 (29.6‐38.0)		
Punjabi	127	60.41 (51.9‐68.92)	39.58 (31.08‐48.08)	0.5734	.448913
Pathan	113	71.82 (63.53‐80.11)	28.18 (19.89‐36.47)	0.3812	.536946
Sindhi	61	82.5 (72.97‐92.03)	17.5 (7.97‐27.03)	4.1637	.041299
Balochi	62	61.11 (48.99‐73.23)	38.88 (26.76‐51.0)	0.3506	.553765
Seraiki	67	52.77 (40.84‐64.7)	47.22 (35.29‐59.15)	2.4063	.120848
Urdu	60	74.67 (63.68‐85.66)	25.32 (14.33‐36.31)	0.6069	.435972

**Table 3:** CYP2B6*6 (rs3745274) genotype frequencies in the Pakistani population

CYP2B6*6	Genotype	N	Observed genotype frequency (CI)	Expected genotype count by HW law	Chi‐Square Statistic	*p*‐value
Pakistani	GG	238	48.57 (44.14‐53.0)	214.89	21.7693	<.05
	GT	173	35.23 (31.0‐39.46)	219.20		
	TT	79	16.19 (12.93‐19.45)	55.89		
Punjabi	GG	58	45.83 (37.19‐54.47)	46.08	19.4987	<.05
	GT	37	29.16 (21.28‐37.04)	60.83		
	TT	32	25 (17.49‐32.51)	20.08		
Pathan	GG	59	51.84 (42.65‐61.03)	58.78	0.0105	>.05
	GT	45	40.05 (31.04‐49.06)	45.43		
	TT	9	8.11 (3.09‐13.13)	8.78		
Urdu	GG	30	50.06 (37.43‐62.69)	26.66	3.7500	>.05
	GT	20	33.33 (21.42‐45.24)	26.66		
	TT	10	16.60 (7.25‐26.07)	6.66		
Seraiki	GG	15	22.22 (12.28‐32.16)	18.80	3.4908	>.05
	GT	41	61.11 (49.46‐72.76)	33.38		
	TT	11	16.66 (7.75‐25.57)	14.80		
Balochi	GG	31	50.00 (37.58‐62.44)	23.29	17.0317	<.05
	GT	14	22.22 (11.89‐32.55)	29.42		
	TT	17	27.77 (16.64‐38.9)	9.29		
Sindhi	GG	43	69.74 (58.23‐81.25)	41.80	1.1482	>.05
	GT	15	25.17 (14.3‐36.04)	17.38		
	TT	03	5.09 (0‐10.6)	1.80		

### 3.2 Alleleic and genotype frequency of CYP2B6*4

Frequencies of CYP2B6*4 alleles in the Pakistani population are shown in Table 4. The frequency of minor alleles for this polymorphism was found to be 25.81% in the Pakistani population (Table 4). In Sindhi and Baloch ethnic populations, the major allele was found at a similar frequency. In the Pathan population, the frequency of minor allele was found to be the highest at 30.22%. The frequency of wild‐type genotype (AA) was 59.79%, AG was 28.77%, and GG was 11.42% in the Pakistan population. Punjabi and Pathan populations showed the highest frequency of wild‐type genotype. The highest prevalence of heterozygous genotype (AG) was found in the Pathan population at 37.16%. Ethnic Baloch Population displayed the highest frequency of homozygous GG genotype. All other ethnic groups also showed a prevalence of GG genotype, albeit at varying rates. (Table 5).

**Table 4:** CYP2B6*4 (rs2279343) allele frequencies in the Pakistani population

Ethnicity	No	Allele A % (CI)	Allele G % (CI)	Chi‐Square Statistic	*p*‐value
Pakistan	490	74.18 (70.31‐78.05)	25.81 (21.94‐29.68)		
Punjabi	127	70.63 (62.71‐78.55)	29.36 (21.44‐37.28)	1.4237	.232800
Pathan	113	69.77 (61.3‐78.24)	30.22 (21.75‐38.69)	1.7161	.190202
Sindhi	61	75.33 (64.51‐86.15)	24.66 (13.84‐35.48)	0.0855	.770022
Balochi	62	74.91 (64.12‐85.7)	25.09 (14.3‐35.88)	0.0384	.844663
Seraiki	67	78.24 (68.36‐88.12)	21.75 (11.87‐31.63)	1.0866	.297231
Urdu	60	82.46 (72.84‐92.08)	17.54 (7.92‐27.16)	3.9531	.046786

**Table 5:** CYP2B6*4 (rs2279343) genotype frequencies in the Pakistani population

CYP2B6*4	Genotype	*n*	Observed genotype frequency (CI)	Expected genotype counts by HW law	Chi‐Square Statistic	*p*‐value
Pakistani	AA	293	59.79 (55.45‐64.13)	269.6577	30.3171	<.05
	AG	141	28.77 (24.76‐32.68)	187.6847		
	GG	56	11.42 (8.6‐14.24)	32.6577		
Punjabi	AA	68	53.54 (44.87‐62.21)	63.7795	3.2904	>.05
	AG	44	34.64 (26.36‐42.92)	52.4409		
	GG	15	11.81 (6.2‐17.42)	10.7795		
Pathan	AA	58	51.32 (42.1‐60.54)	55.2301	1.5345	>.05
	AG	42	37.16 (28.25‐46.07)	47.5398		
	GG	13	11.50 (5.62‐17.38)	10.2301		
Urdu	AA	44	73.33 (62.14‐84.52)	40.8375	7.997	<.05
	AG	11	18.33 (8.54‐28.12)	17.325		
	GG	5	8.33 (1.34‐15.32)	1.8375		
Seraiki	AA	46	68.65 (57.54‐79.76)	41.1381	12.2684	<.05
	AG	13	19.40 (9.93‐28.27)	22.7239		
	GG	8	11.94 (4.18‐19.7)	3.1381		
Balochi	AA	40	64.51 (52.6‐76.42)	34.875	12.0502	<.05
	AG	13	20.96 (10.83‐31.09)	23.25		
	GG	9	14.51 (5.74‐23.28)	3.875		
Sindhi	AA	37	60.65 (48.39‐72.91)	34.6885	2.5472	>.05
	AG	18	29.50 (18.06‐40.94)	22.623		
	GG	6	9.83 (2.36‐17.3)	3.6885		

### 3.3 Alleleic and genotype frequency of CYP2B6*3

Frequencies of CYP2B6*3 alleles in the Pakistani population are shown in Table 6. The frequency of the major allele was 93.5% and of minor allele was 6.5%. The major allele was found slightly more prevalent in Baloch and Pathan ethnic groups at 95.87% and 94.38%, respectively, compared to Sindhi, Punjabi, Seraiki, and Urdu ethnicities, where the prevalence of minor allele was slightly higher (Table 6). The frequency of CC genotype was 90.20%, AC was 6.73%, and AA was 3.06% in the Pakistan population. The ethnic Baloch Population showed a higher frequency of wild‐type genotype (CC) at 93.54% while Sindhi, Pathan, Urdu, and Seraiki ethnicities had a lower prevalence of wild‐type genotype. Urdu Population showed the highest frequency of homozygous genotype (AA) at 5% (Table 7).

**Table 6:** CYP2B6*3 (rs45482602) allele frequencies in the Pakistani population

Ethnicity	no	Allele C % (CI)	Allele A % (CI)	Chi‐Square Statistic	*p*‐value
Pakistan	490	93.5 (91.3‐95.7)	6.5 (4.3‐8.7)		
Punjabi	127	92.34 (87.7‐97.0)	7.65 (3.0‐12.3)	0.5739	.448727
Pathan	113	94.38 (90.13‐98.63)	5.61 (1.37‐9.85)	0.1862	.666126
Sindhi	61	91.67 (84.74‐98.6)	8.32 (1.39‐15.25)	0.4808	.488068
Balochi	62	95.87 (90.92‐100)	4.12 (0‐9.07)	1.1726	.278876
Seraiki	67	93.26 (87.26‐99.26)	6.73 (0.73‐12.73)	0.0066	.935026
Urdu	60	92.81 (86.27‐99.35)	7.18 (0.65‐13.71)	0.1621	.687190

**Table 7:** CYP2B6*3 (rs45482602) genotype frequencies in Pakistani population

CYP2B6*3	Genotype	*n*	Observed genotype frequency (CI)	Expected genotype count by HW law	Chi‐Square Statistic	*p*‐value
Pakistani	CC	442	90.20 (87.57‐92.83)	429.025	94.9518	<.05
	AC	33	6.73 (4.51‐8.95)	58.95		
	AA	15	3.06 (1.54‐4.58)	2.025		
Punjabi	CC	114	89.76 (84.49‐95.03)	110.5689	23.7686	<.05
	AC	9	7.08 (2.62‐11.54)	15.8622		
	AA	4	3.14 (0.11‐6.17)	0.5689		
Pathan	CC	103	91.15 (85.91‐96.39)	100.3739	20.7651	<.05
	AC	7	6.19 (1.75‐10.63)	12.2522		
	AA	3	2.65 (0‐5.61)	0.3739		
Urdu	CC	54	90 (82.41‐97.59)	51.3375	24.5483	<.05
	AC	3	5 (0‐10.51)	8.325		
	AA	3	5 (0‐10.51)	0.3375		
Seraiki	CC	60	89.55 (82.23‐96.87)	58.3022	10.9595	<.05
	AC	5	7.46 (1.17‐13.75)	8.3955		
	AA	2	2.98 (0‐7.05)	0.3022		
Balochi	CC	58	93.54 (87.42‐99.66)	57.1008	8.709	<.05
	AC	3	4.83 (0‐10.17)	4.7984		
	AA	1	1.61 (0‐4.74)	0.1008		
Sindhi	CC	53	86.88 (78.41‐95.35)	51.4098	7.3208	<.05
	AC	6	9.83 (2.36‐17.3)	9.1803		
	AA	2	3.27 (0‐7.73)	0.4098		

## 4 DISCUSSION

According to its Statistics Bureau, Pakistan, with an estimated population of over 210 million, is the sixth most populous country in the world (Pakistan Bureau of Statistics, 2017). The country has a young, multi‐ethnic, and multi‐cultural society and despite being home to a vast population, pharmacogenetic studies on how its population responds to various pharmaceutical drugs are rare. The largest ethnic group in Pakistan is Punjabis, which makes up about 38.78% of the population, followed by Pashtuns (18.24%), Sindhis (14.57%), Seraikis (10.53%), Urdu speaking (7.57%), and Baloch (3.57%) (Taus‐Bolstad, 2003). These ethnic groups represent about 94% of the Pakistani population. Genetic variations in CYP genes affecting the metabolism of xenobiotics and drug response have not been investigated in these ethnic groups. Our study partly addresses this issue by reporting frequencies of the three of the most important single‐nucleotide polymorphisms in the *CYP2B6* gene.

The frequencies of different *CYP2B6* polymorphisms have been studied in diverse populations, showing a highly variable distribution (Arnaldo et al., 2013). Specifically, for the CYP2B6*6 polymorphism, the global distribution for the G and T alleles is 73 and 26%, respectively (genotypes GG: 54.1%, GT: 38.4%, TT: 7.5%). The frequency of CYP2B6*6 minor allele (T) was reported at about 23.6% from Europe, 37.4% from Africa, 37.3% in America, 21.5% in East Asians, while in the South Asian region its prevalence is estimated to be 38.1% (Auton et al., 2015) (Table 8). However, in the Pakistani population, we found its prevalence at 33.8%. This means that our investigation shows a slightly lower prevalence of this allele. Similar variations have also been noted previously in other populations. For example, the frequency of the genotype variants for CYP2B6*6 in the Argentinian Population was found to be 10.8% (for TT genotype) (Scibona et al., 2015) is double than its frequency in European populations (4.2%) and similar to the frequency found in Native Americans and persons of African descent (13.3 and 13%, respectively). In our study, the frequency of CYP2B6*6 minor allele (T) was highest in the Seraiki ethnicity. Punjabi and Baloch populations reported this variant at a slightly higher frequency than observed for the whole Pakistani population. Sindhi Population showed the highest prevalence of wild‐type allele and the lowest frequency of the minor allele. These results suggest that a significant portion of the Pakistani population may experience unexpected therapeutic and adverse effects of drugs metabolized chiefly by the CYP2B6 enzyme.

**Table 8:** CYP2B6*6, *4, and *3 allele frequencies as observed in various superpopulations in the 1000 Genome population

Population	CYP2B6*6 (rs3745274)		CYP2B6*4 (rs2279343)		CYP2B6*3 (rs45482602)	
	G	T	A	G	C	A
AFR	0.626	0.374	0.829	0.129	0.995	0.005
AMR	0.627	0.373	0.834	0.166	0.995	0.005
EAS	0.785	0.215	0.853	0.147	1.000	0.000
EUR	0.764	0.236	0.912	0.088	0.998	0.002
SAS	0.619	0.381	0.748	0.252	0.998	0.002

Abbreviations: AFR, African; AMR, American; EAS, East Asian; EUR,European; SA, South Asian.

The frequency of CYP2B6*4 minor allele (G) from the African Population is reported at 12.9%, from America at 16.6%, and East Asia at 14.7%. The lowest frequency of this variant is reported from Europe at 8.8%, while the South Asian region was reported to display the highest frequency of this allele at 25.2% (Auton et al., 2015). In the Pakistani Population, this allele was found in the same range (25.81%). The frequency of CYP2B6*4 minor allele (G) was highest in the Pathan population followed by the Punjabi Population. Sindhi, Baloch, and Seraiki populations reported this variant at the same rate observed for the whole Pakistani population. Urdu speaking population showed the highest prevalence of wild‐type alleles among Pakistani ethnicities and the lowest frequency of the minor allele.

The frequency of CYP2B6*3 minor allele (A), as reported previously from various regions of the world, is about 2% from Europe, 5% from Africa, 5% in America. In contrast, this allele is not reported from the East Asian region. However, in the Pakistani population, its frequency is reported at 6.5% (Auton et al., 2015). The difference in allele and genotype frequencies between other populations and this study may be since our study estimated the frequencies in six different ethnicities while in the 1000 Genome project, the Pakistani population is represented by one ethnicity only. The difference in the sample size may be another reason for the discrepancy. Among various Pakistani ethnic groups, the frequency of CYP2B6*3 minor allele (A) was highest in the Sindhi Population. Punjabi, Pathan, Seraiki, and Urdu populations reported this variant at roughly the same rate observed for the whole Pakistani population. Baloch Population showed the highest prevalence of wild‐type allele and the lowest frequency of the minor allele.

Our results are largely in agreement with earlier studies reporting *CYP2B6* polymorphisms (Auton et al., 2015; Scibona et al., 2015). However, some small differences in the frequencies of minor alleles are observed. CYP2B6*6 was present at a slightly lower frequency than the South Asian Population (33.8% vs. 38.1%), while CYP2B6*3 and *4 were present at slightly higher frequencies than the South Asian Population (25.8% vs. 25.2% for CYP2B6*4 and 6.5% vs. 0.02% for CYP2B6*3). Taken together, these findings suggest that important *CYP2B6* polymorphisms are present in high enough frequency in the Pakistani population to warrant more studies on individual drugs that are metabolized by CYP2B6 enzyme. The effects of these polymorphisms on individual drugs such as methadone, bupropion, cyclophosphamide, efavirenz, etc. would be important to investigate.

To our knowledge, this is the first study to report frequencies of *CYP2B6* gene polymorphisms in various ethnicities of the Pakistani population. Genetic information about patients’ *CYP2B6* gene is likely to help physicians prescribe to patients the most suitable and safest drug based on their genetic make‐up. With roughly 7.2% clinically available drugs metabolized by CYP2B6 enzyme(Zanger & Schwab, 2013) and a significant fraction of the Pakistani population having low activity alleles, the number of patients affected by these genetic variations is substantial. We propose carrying out further studies with individual drugs metabolized by CYP2B6 to shed more light on genotype–phenotype relations.

## CONFLICT OF INTEREST

The authors declare that they have no competing interests.

## AUTHORS’ CONTRIBUTIONS

SA and SK conceptualized the study. AUK and II searched the literature, collected the data, and helped in manuscript preparation. SA and KJ helped prepare the manuscript. SA, SK, and KJ refined the manuscript for publication. All authors read and approved the final manuscript for publication.

## ETHICS STATEMENT

This study was approved by the Institutional Review Board and Ethics Committee of Shifa International Hospital and Shifa Tameer‐e‐Millat University, Islamabad, Pakistan.
